# Severity of traumatic brain injury correlates with long-term cardiovascular autonomic dysfunction

**DOI:** 10.1007/s00415-017-8581-1

**Published:** 2017-08-02

**Authors:** Max J. Hilz, Ruihao Wang, Jörg Markus, Fabian Ammon, Katharina M. Hösl, Steven R. Flanagan, Klemens Winder, Julia Koehn

**Affiliations:** 1 Clinical Department of Autonomic Neurology, University College London, Institute of Neurology and The National Hospital for Neurology and Neurosurgery, Queen Square, London, WC1N 3BG UK; 2 Department of Neurology, Friedrich-Alexander-Universität Erlangen-Nürnberg (FAU), Erlangen, Germany; 3 Icahn School of Medicine at Mount Sinai, Department of Neurology, New York, NY USA; 4 Department of Psychiatry and Psychotherapy, Paracelsus Medical University, Nuremberg, Germany; 5 Department of Rehabilitation Medicine, New York University School of Medicine, New York, NY USA

**Keywords:** Orthostatic challenge, Traumatic brain injury, Autonomic dysfunction, Differences in severity

## Abstract

After traumatic brain injury (TBI), central autonomic dysfunction might contribute to long-term increased mortality rates. Central autonomic dysfunction might depend on initial trauma severity. This study was performed to evaluate differences in autonomic modulation at rest and upon standing between patients with a history of mild TBI (post-mild-TBI patients), moderate or severe TBI (post-moderate–severe-TBI patients), and healthy controls. In 20 post-mild-TBI patients (6–78 months after TBI), age-matched 20 post-moderate–severe-TBI patients (6–94 months after TBI) and 20 controls, we monitored respiration, RR intervals (RRI) and systolic blood pressure (BPsys) at supine rest and upon standing. We determined mainly sympathetic low (LF) and parasympathetic high (HF) frequency powers of RRI fluctuations, sympathetically mediated LF-BPsys powers, LF/HF-RRI ratios, normalized (nu) LF-RRI and HF-RRI powers, and compared data between groups, at rest and upon standing (ANOVA with post hoc testing). We correlated autonomic parameters with initial Glasgow Coma Scale (GCS) scores (Spearman test; significance: *p* < 0.05). Supine BPsys and LFnu-RRI powers were higher while HFnu-RRI powers were lower in post-moderate–severe-TBI patients than post-mild-TBI patients and controls. LFnu-RRI powers were higher and HFnu-RRI powers were lower in post-mild-TBI patients than controls. Upon standing, only post-mild-TBI patients and controls increased LF-BPsys powers and BPsys and decreased HF-RRI powers. GCS scores correlated positively with LFnu-RRI powers, LF/HF-RRI ratios, and inversely with HFnu-RRI powers, at standing position. More than 6 months after TBI, there is autonomic dysfunction at rest and upon standing which is more pronounced after moderate–severe than mild TBI and in part correlates with initial trauma severity.

## Introduction

Patients with a history of mild traumatic brain injury (TBI) frequently have long-lasting post-traumatic complications including neurological [33], neuro-psychological [10], and social [27] sequelae, and even an increased risk of unexplained long-term mortality [7, 34–36].

Particularly, the cause of increased long-term mortality rates [34, 35] is unknown but has been considered to be associated with subtle impairment of the central network assuring autonomic regulation [4, 19, 20, 49, 50].

In patients with no clinically overt autonomic dysfunction but with a history of mild TBI [19, 20], we found a decrease in the overall cardiovascular modulation at rest with a shift towards more sympathetic than parasympathetic activity and a decrease in baroreflex-mediated sympathetic and parasympathetic responses to orthostatic challenge [20]. Similarly, we found a compromised ability to activate cardiovagal outflow upon eyeball pressure stimulation, which slows heart rate independently from the baroreflex arc [19]. These findings support the assumption [4] that minor central autonomic network (CAN) dysfunction accounts for the subclinical cardiovascular dysregulation seen in patients even years after mild TBI [1, 4, 19, 20].

In patients with moderate or severe TBI, autonomic dysfunction, so far, has only been reported during the acute phase after the trauma [16, 17, 39, 51]. In acute brain injuries, Goldstein and co-workers found evidence for cardiovascular and autonomic uncoupling and a deterioration of heart rate and blood pressure variability with increasing trauma severity [16]. Papaioannou et al. showed that mortality of acute brain injury patients is linked with decreased heart rate variability and baroreflex sensitivity [39]. Hendricks et al. observed episodes of dysautonomia in 11.8% of patients during the acute phase of severe TBI and found associations between dysautonomia and diffuse axonal injury as well as spasticity at follow-up [17]. Similarly, Sykora and co-workers recently reported associations between autonomic cardiovascular dysfunction and increased mortality in patients with acute, severe traumatic brain injury [51].

Some studies also suggest that long-term mortality rates after TBI increase with trauma severity [12, 13]. Based on the above findings regarding correlations between TBI severity and increasing autonomic impairment during the acute trauma phase [16, 39, 51], and on the assumption that CAN dysfunction contributes to cardiovascular autonomic dysregulation in patients with a history of mild TBI, we hypothesize that patients with a history of more severe, i.e., with moderate or severe TBI also have more prominent CAN alteration and consequently retain more prominent cardiovascular autonomic dysregulation than do patients with a history of mild TBI.

However, at least two studies showed that the risk of long-term mortality does not differ between patients who had experienced a mild, moderate or severe TBI provided more than 6 [7] or 12 months [34] have passed since the trauma. If central autonomic dysfunction contributes to the increased risk of long-term mortality, similar mortality rates reported in patients with different severity of TBI after more than 6 or 12 months [7, 34] might suggest that autonomic function or dysfunction does not differ between patients who have experienced mild TBI and those who had moderate or severe TBI more than 6 months ago.

While it is unclear whether autonomic dysfunction is more prominent with a history of more severe TBI or does not differ between patients who experienced different TBI severity more than 6 months ago, this issue may be of prognostic and therapeutic relevance. Therefore, we evaluated whether patients with a history of moderate or severe TBI have a more prominent alteration in cardiovascular autonomic modulation at rest and upon orthostatic challenge than do patients with a history of mild TBI.

## Materials and methods

We studied heart rate, blood pressure and autonomic responses to active standing-up in 20 patients (6 women, 14 men, mean age 33.1 ± 13.5 years) who had suffered a mild TBI 6–78 months prior to examination (mean post-injury interval 25.2 months; standard deviation (SD) 20.5 months) and in 20 patients (4 women, 16 men, mean age 34.5 ± 10.0 years) who had suffered a moderate or severe TBI (moderate–severe TBI) 6–94 months prior to examination (mean post-injury interval 35.9 months; SD 27.7 months). Patients were asked to participate in the assessment of autonomic function after we had retrospectively evaluated their medical records, physical and neurological status and TBI severity at the time of the initial trauma. From the medical records, we assessed whether any spinal trauma was associated with the TBI or whether decompression therapy had to be performed at the time of the acute injury. The structural TBI severity was assessed via computed tomography (CT) scans which also identified possible traumatic subarachnoid hemorrhage.

We used the Mayo Clinic Classification for TBI severity to define TBI severity [32] as it is well suited for retrospectively determining TBI severity [32]. Mild TBI was diagnosed if one or more of the following criteria applied: (1) loss of consciousness of momentary to less than 30 min; (2) post-traumatic anterograde amnesia of momentary to less than 24 h; (3) depressed, basilar or linear skull fracture (dura intact). Moderate or severe TBI (moderate–severe TBI) was diagnosed if one or more of the following criteria applied: (1) death due to this TBI; (2) loss of consciousness of more than 30 min; (3) post-traumatic anterograde amnesia of more than 24 h; (4) worst Glasgow Coma Scale score within the first 24 h of less than 13 (unless, e.g., attributable to intoxication, sedation, systemic shock); (5) more than one of the following was present: intracerebral, subdural or epidural hematoma, cerebral or hemorrhagic contusion, dura penetration, subarachnoid hemorrhage, brain stem injury [32]. To assure that any signs of autonomic dysfunction cannot be related to drugs, medication or other diseases affecting the autonomic nervous system, we excluded patients from the study in whom the TBI had occurred due to drugs, alcohol, or medications [23]. In addition, we excluded persons with a history of diseases possibly affecting autonomic regulation such as diabetes, arterial hypertension, cardiac arrhythmias, ischemic heart disease or chronic heart failure, and patients on medication affecting autonomic regulation, e.g., antihypertensive drugs.

Clinical outcome at the time of autonomic testing was assessed by means of Extended Glasgow Outcome Scores (GOSE). We intended to determine whether there was any subtle cardiovascular dysregulation in patients who had a history of mild or moderate–severe TBI but had no clinically overt signs or symptoms of cardiovascular autonomic dysfunction in TBI patients. Therefore, we only enrolled patients who had well recovered after the TBI and had reached a score of at least 6 on the GOSE reflecting independence in activities of daily living but reduced capacity for former work [53], and who had no signs or symptoms of clinically overt autonomic dysfunction at the time of autonomic testing.

Findings in patients were compared to those of 20 age- and sex-matched healthy volunteers (6 women, 14 men, mean age 29 ± 10 years).

The Institutional Review Board (IRB) at the New York University and the ethics committee of the University of Erlangen-Nuremberg, Germany, had approved the study and written informed consent was obtained from all participants according to the declaration of Helsinki (2000) of the World Medical Association.

Participants were tested between 9 a.m. and 2 p.m. in a reclining armchair, in a quiet room with 24 °C ambient temperature and stable humidity. Before testing, a resting period of at least 40 min was used to familiarize participants with our equipment and testing procedures, and to assure cardiovascular resting stability.

Using a 3-lead electrocardiogram (ECG), we monitored electrocardiographic RR intervals (RRI). Systolic and diastolic beat-to-beat blood pressures (BPsys, BPdia) were recorded continuously at the left hand, using finger pulse photoplethysmography (Portapres; TPD BMI) [18]. Respiratory frequency (RESP) was recorded using a piezoelectric respiratory belt attached to the lower thorax [18]. Data were sampled on a custom designed data acquisition and analysis system (SUEmpathy™, SUESS GmbH, Germany) for off-line analysis.

As the patients had no clinical signs or symptoms of overt autonomic dysfunction, we evaluated the initial 30 s of baroreflex responses to standing-up since this phase shows the most rapid and pronounced changes in cardiovascular autonomic parameters [20, 58].

From 2-min recordings at rest, we calculated mean values and standard deviation of all bio-signals and assessed autonomic parameters. We compared these supine values with parameters recorded during the initial 30 s of 10-min standing. We chose the initial phase of cardiovascular adaptation to standing as the prominent autonomic responses during the first 30–60 s of standing seemed more likely to unveil subtle autonomic impairment in patients without overt signs or symptoms of autonomic dysfunction than later phases of adjustment to orthostatic challenge [18, 20, 52, 55, 58]: baroreflex activation upon standing induces an abrupt inhibition of cardiac vagal tone with a peak occurring already approximately 3 s after standing up [58]. After approximately 5 s of standing, there is a more gradual secondary increase in heart rate due to continued parasympathetic withdrawal and increase in sympathetic tone, resulting in a secondary heart rate peak at around 12 s of standing. After approximately 30 s of standing, blood pressure and heart rate recovery is already complete in healthy persons.

As the initial phase of autonomic adjustment to standing-up is particularly suited to evaluate baroreflex function [18, 52, 57], we calculated mean values and standard deviation of all bio-signals during the initial 30 s after standing-up.

To assess heart rate variability at rest and during active standing, we determined the standard deviation (SD) and coefficient of variation (CV) of RRIs, both reflecting sympathetic and parasympathetic heart rate modulation [18, 52], and the square root of the mean squared differences of successive RRIs (RMSSD) reflecting parasympathetic influences on heart rate variability [18, 52]. As a measure of baroreflex-induced heart rate responses to active standing, we assessed the so-called RRI-30:15 ratio reflecting the degree of heart rate changes upon standing-up by calculating the ratio between the shortest RR interval at or around the 15th heart beat and the longest RR interval at or around the 30th heart beat after standing up.

RRI and BP values show slow underlying fluctuations that are largely mediated by undulating activity of the autonomic nervous system. For spectral analysis of slow sympathetically and parasympathetically mediated RRI and BP oscillations, we used trigonometric regressive spectral analysis (TRS) of 30 s epochs that were re-analyzed every 5 s resulting in epoch-overlaps of 25 s and thus assuring continuous monitoring of sympatho-vagal activity. The TRS analysis is suited to evaluate changes in oscillations of bio-signals as short as 25–30 s [22, 42, 44, 59]. As the TRS algorithm is based on the sequential analysis of very short signal epochs which advances beat by beat along longer bio-signal time series [44, 59], the analysis provides a high temporal resolution of frequencies and amplitudes of signal oscillations occurring within a data segment [44, 59]. Thus, the TRS algorithm is suited to analyze both stationary and non-stationary signals [20, 44, 59]. Therefore, TRS analysis is best suited for our study.

RRI oscillations in the so-called high-frequency range (HF: 0.15–0.5 Hz) are mediated by changes in parasympathetic activity, while BP oscillations in the HF-range are mainly a mechanical consequence of respiration-induced increases in venous return and stroke volume. BP oscillations in the so-called low-frequency range (LF: 0.04–0.15 Hz) are mediated by changes in sympathetic activity while LF oscillations of RRI are not only due to sympathetic outflow but also contain parasympathetically mediated oscillations, particularly in supine position.

We determined powers of sympathetic and parasympathetic influences on RRI and BP variability by quantifying LF and HF components of signal variability as integral under the power spectral density curves [18, 52].

To adjust for differences in overall signal modulation between patients and healthy volunteers and between resting and standing conditions, we normalized LF and HF powers of RRI [18, 52] by calculating percentage values of LF and HF powers in relation to powers of RRI fluctuation in the entire range from 0.04 Hz to 0.5 Hz, with LFnu-RRI = [LF/(LF + HF)] × 100%, and HFnu-RRI = [(HF/(LF + HF)] × 100% [38]. To further adjust for differences between groups and positions, we calculated the ratio between RRI oscillations in the LF and HF ranges, and used the LF/HF ratio of RRI as index of the balance between sympathetic and vagal influences on heart rate modulation [38, 52].

To determine baroreflex sensitivity (BRS), the TRS software selected pairs of LF and HF oscillations of systolic BP and RRI with high coherence [26]. Coherence spans from 0, i.e., no association, to 1, i.e., maximum association [8]. High coherence at a specific frequency, e.g., >0.7, indicates a stable phase relation—and thus synchronization—between two signals oscillating at this frequency [8]. Then, the sensitivity of the baroreflex loop (ms mmHg^−1^) can be derived as gain values from changes in RRIs (ms) in relation to changes in systolic BP (mmHg) [43].

To evaluate whether there is an association between GCS scores or the interval since trauma and autonomic function, we determined the correlation between these two values with autonomic parameters.

### Statistical analysis

We used the Kolmogorov–Smirnov test to test for normal distribution of data. Normally distributed data were presented as mean ± standard deviation (SD).

Differences in cardiovascular parameters between healthy participants, mild-TBI patients, and moderate–severe TBI patients were evaluated by analysis of variance for repeated measurements (ANOVA, general linear model), with “maneuver” (rest and standing) as within subject factor and “group” (mild-TBI patients, moderate–severe TBI patients, and controls) as between-subject factor. The suitability of the ANOVA model was assessed by Mauchly’s test of sphericity. In case of violation of the sphericity assumption, the Greenhouse–Geisser correction was employed. In cases of significant ANOVA results, post hoc single comparisons were performed. In case of normally distributed data, we used t tests for unpaired samples for comparison between groups and *t* tests for paired samples for comparison of values at rest and during standing.

In case of non-normally distributed data, we used two-sided Mann–Whitney-*U*-tests to compare parameters between groups and two-sided Wilcoxon tests to compare parameters at rest and during standing.

We correlated bio-signals and autonomic parameters with the severity of the TBI, as assessed by the Glasgow Coma Scale (GCS) scores at the time of the injury, and with the interval between trauma and autonomic testing, using the Spearman rank correlation test for non-normally distributed data and the Pearson test for normally distributed data.

Significance was set at *P* < 0.05. A commercially available statistical program (IBM SPSS Statistics for Windows, Version 20.0, Armonk, NY, USA) was used for data analysis.

## Results

There was no significant age difference between patients with a history of mild TBI (33.1 ± 13.5 years), patients with a history of moderate–severe TBI (34.5 ± 10.0 years) and healthy controls (29.3 ± 11.4 years, *P* > 0.05). Moreover, the interval between trauma and autonomic testing did not differ significantly between mild TBI (25.2 ± 20.5 months) and moderate–severe TBI patients (35.9 ± 27.7 months, *P* = 0.265).

One patient with moderate–severe TBI had required decompression therapy at the time of the initial hospital admission, and two patients with moderate–severe TBI had suffered a traumatic subarachnoid hemorrhage. None of our TBI patients had an associated spinal trauma.

At the time of our autonomic testing, 4 of the 20 patients with a history of mild TBI had GOSE values of 7 (reflecting near complete recovery), the other 16 patients had GOSE values of 8 (reflecting complete recovery) [17, 53]. Four of the 20 patients with a history of moderate–severe TBI had GOSE values of 6; five patients had GOSE values of 7, and 11 patients had GOSE values of 8.

**Fig. 1:**
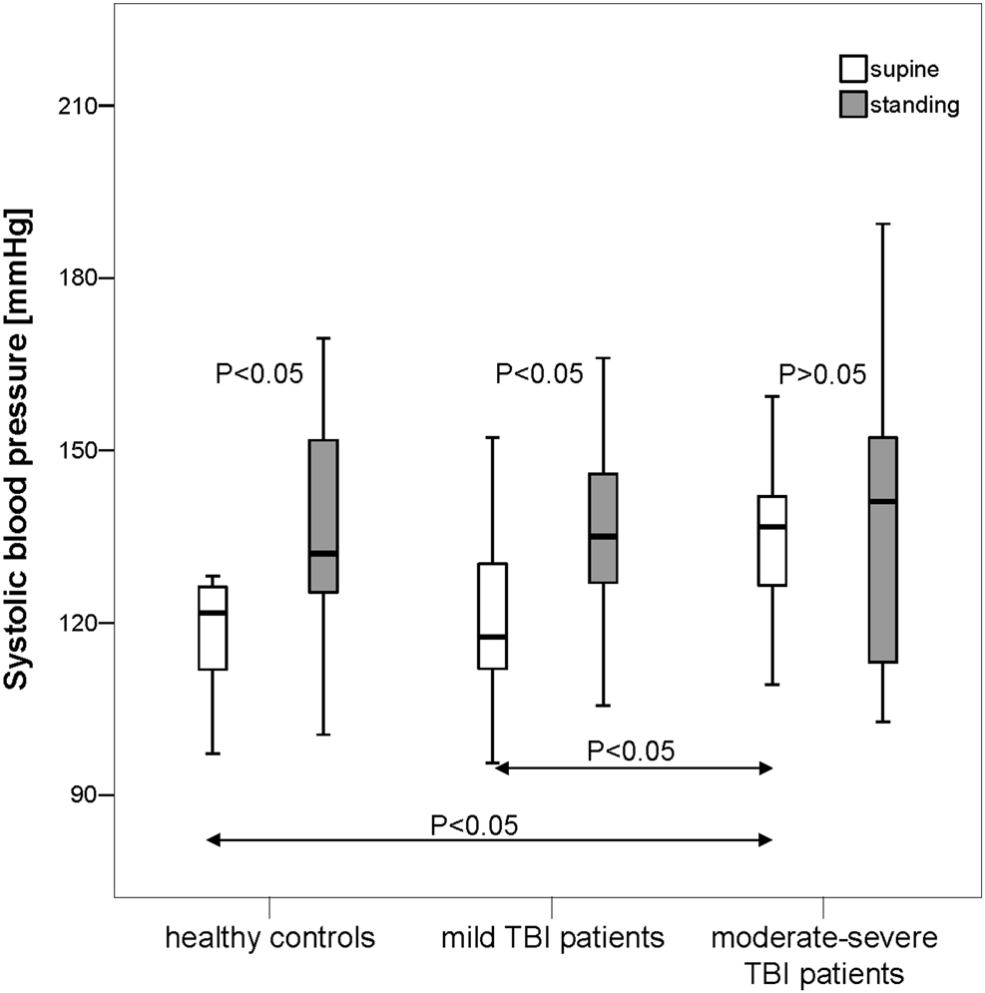
Systolic blood pressure (BPsys) in supine and standing positions in 20 patients with a history of mild TBI, 20 patients with a history of moderate or severe TBI (moderate–severe TBI) and 20 healthy controls. In supine position, BPsys was significantly higher in moderate–severe TBI patients than in mild-TBI patients or controls (*P* < 0.05). Upon standing, healthy controls and mild-TBI patients significantly increased BPsys while moderate–severe TBI patients did not change their BPsys. Data are presented as *box plots*. The *top* of the *box* represents the 75th percentile (*upper quartile*), the *bottom* of the *box* represents the 25th percentile (*lower quartile*), the *line* in the *middle* represents the 50th percentile (median). The *ends* of the *whiskers* represent the *highest* and *lowest values* that are not outliers or extreme values. *White boxes* illustrate supine values, and gray *boxes* illustrate standing values

At supine rest, BPsys was significantly higher in moderate–severe TBI patients than in mild-TBI patients or controls, while all three groups had similar BPdia, RRIs, RESP, RRI-SDs, RRI-CVs, RMSSD, LF-RRI powers, LF-BPsys and HF-BPsys powers, and BRS values (*P* > 0.05) (Fig. 1).

**Fig. 2:**
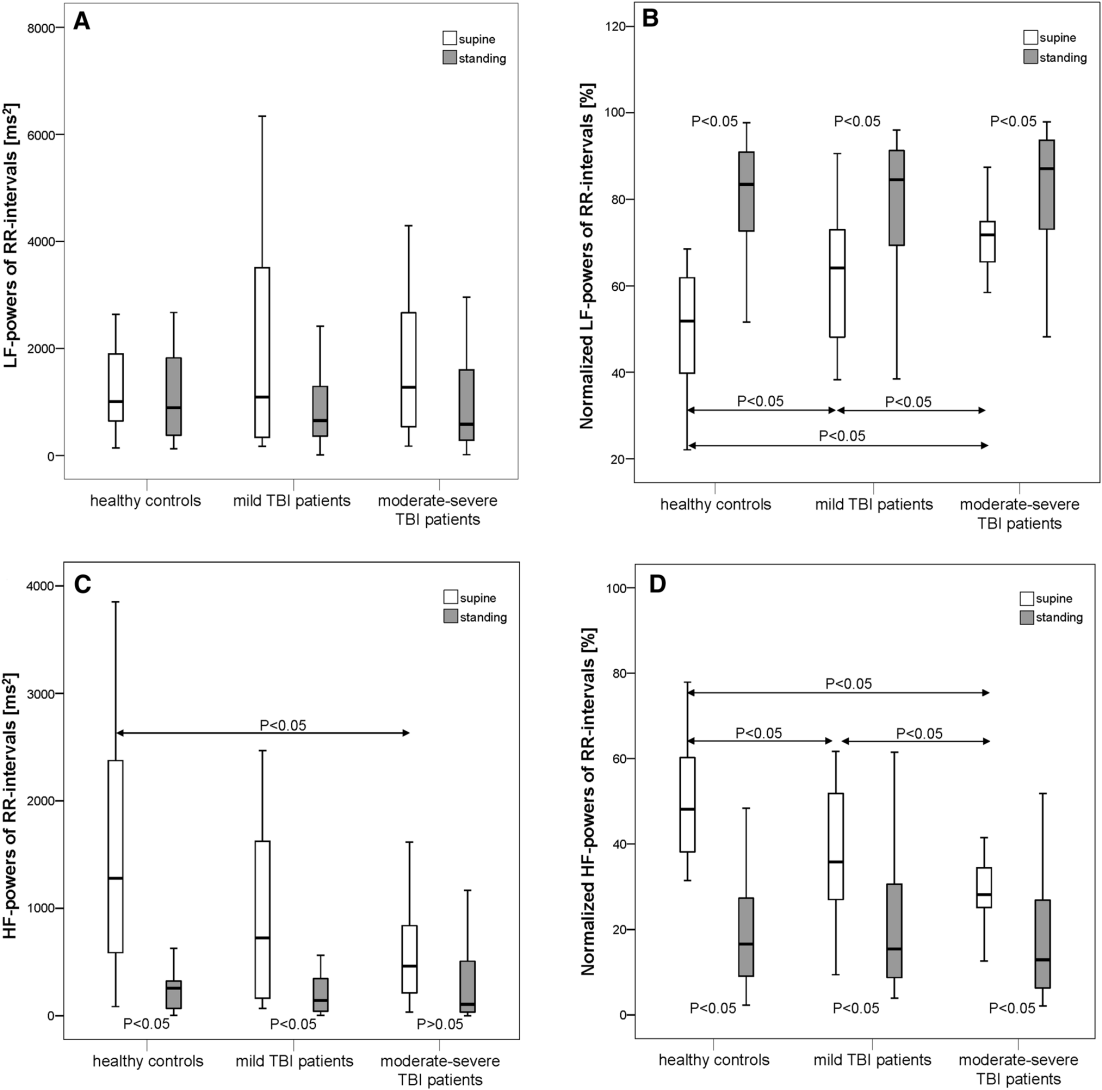
Absolute and normalized low-frequency powers of RR intervals (LF-RRI) and high-frequency powers of RR intervals (HF-RRI) in supine and standing positions in 20 patients with a history of mild TBI, 20 patients with a history of moderate or severe TBI (moderate–severe TBI) and 20 healthy controls. At supine rest, normalized RRI-LF powers (LFnu-RRI powers) were significantly higher while normalized RRI-HF powers (HFnu-RRI powers) were significantly lower in the moderate–severe TBI patients group than in the patients with a history of mild TBI or the healthy controls (*P* < 0.05). In mild-TBI patients, LFnu-RRI powers again were significantly higher and HFnu-RRI powers were lower than in controls. In moderate–severe TBI patients, the parasympathetically mediated HF-RRI powers were significantly lower than in healthy controls. Upon standing up, all groups significantly decreased normalized HF-RRI powers, and significantly increased normalized LF-RRI powers, LF-RRI powers did not change in any group (*P* > 0.05). Healthy controls and mild-TBI patients significantly decreased HF-RRI powers, while moderate–severe TBI patients did not change their HF-RRI powers upon standing-up. **a** Absolute LF powers of RR intervals; **b** normalized LF powers of RR intervals; **c** absolute HF powers of RR intervals; **d** normalized HF powers of RR intervals. *Data* are presented as *box plots*. The *top* of the *box* represents the 75th percentile (*upper quartile*), the *bottom* of the *box* represents the 25th percentile (*lower quartile*), the line in the *middle* represents the 50th percentile (median). The ends of the *whiskers* represent the *highest* and *lowest values* that are not outliers or extreme values. *White boxes* illustrate supine values, and *gray boxes* illustrate standing values

Similar to BPsys, sympathetically mediated LFnu-RRI powers were also higher in the moderate–severe TBI patients group while HFnu-RRI powers were significantly lower than in the two other groups (*P* < 0.05; Table 2; Fig. 2), while patients with a history of mild TBI also had higher LFnu-RRI powers and lower HFnu-RRI powers than had the controls (Fig. 2). Parasympathetically mediated HF-RRI powers were also significantly lower (Fig. 2) and LF/HF-RRI ratios were significantly higher in the moderate–severe TBI patients than the healthy controls. In contrast, mild-TBI patients had only slightly though not significantly higher LF/HF-RRI ratios than the healthy controls (*P* = 0.072).

Upon standing up, all groups significantly decreased RRIs, RMSSDs, normalized HF-RRI powers, and BRS (*P* < 0.05), and significantly increased BPdia, normalized LF-RRI powers, LF/HF-RRI ratios, and HF powers of BPsys (*P* < 0.05); while RESP, RRI-CVs and LF-RRI powers remained unchanged (*P* > 0.05). Moreover, healthy controls and mild-TBI patients significantly increased BPsys and significantly decreased HF-RRI powers, while moderate–severe TBI patients did not change their BPsys and HF-RRI powers upon standing-up. Sympathetically mediated LF-BPsys powers only increased significantly in healthy controls, but not in the two patient groups.

During standing, the three groups had similar values for RRIs, BPsys, BPdia, RESP, RRI-CVs, RMSSD, RRI-30:15 ratios, normalized LF and HF powers of RRI, LF and HF powers of BPsys, LF/HF ratios and BRS (*P* > 0.05; Tables 1, 2).

**Table 1:** Mean values ± standard deviation of respiration, systolic and diastolic blood pressure, RR intervals, and time domain parameters of RRI variability in supine and standing positions in 20 patients with a history of mild TBI, 20 patients with a history of moderate or severe TBI (moderate–severe TBI) and 20 healthy controls

Parameter	Position	Healthy controls (*n* = 20)	Mild-TBI patients (*n* = 20)	Moderate–severe TBI patients (*n* = 20)	Mild TBI vs. controls	Moderate–severe TBI vs. controls	Mild TBI vs. moderate–severe TBI
RESP (cpm)	Supine	14.1 ± 3.1	14.6 ± 3.4	14.4 ± 2.6	*P* = 0.391	*P* = 0.545	*P* = 0.736
	Standing	14.5 ± 3.1	13.6 ± 4.1	13.4 ± 3.3	*P* = 0.476	*P* = 0.158	*P* = 0.594
BPsys (mmHg)	Supine	**121.3** **±** **15.2**	**122.5** **±** **16.3**	133.6 ± 14.2	*P* = 0.821	*P* = ***0.012***	*P* = ***0.027***
	Standing	**136.2** **±** 20.5 *	**136.9** **±** 15.1 *	138.6 ± 24.7	*P* = 0.899	*P* = 0.732	*P* = 0.792
BPdia (mmHg)	Supine	**60.5** **±** **8.0**	**61.7** **±** **7.4**	**65.3** **±** **10.2**	*P* = 0.616	*P* = 0.107	*P* = 0.214
	Standing	**72.3** **±** 8.3 *	**70.5** **±** 10.1 *	**73.3** **±** 11.3 *	*P* = 0.542	*P* = 0.754	*P* = 0.418
RRI (ms)	Supine	**1041.0** **±** **166.7**	**943.1** **±** **123.5**	**1038.2** **±** **203.3**	*P* = 0.052	*P* = 0.962	*P* = 0.144
	Standing	**801.0** **±** 131.5 *	**775.6** **±** 132.8 *	**790.4** **±** 197.7 *	*P* = 0.547	*P* = 0.843	*P* = 0.783
RRI-SD (ms)	Supine	59.4 ± 36.5	**50.9** **±** **27.0**	49.5 ± 22.2	*P* = 0.406	*P* = 0.307	*P* = 0.863
	Standing	49.1 ± 25.4	**39.4** **±** 23.0 *	41.3 ± 34.6	*P* = 0.212	*P* = 0.420	*P* = 0.839
RRI-CV (%)	Supine	5.7 ± 3.3	5.7 ± 3.3	4.7 ± 1.6	*P* = 0.687	*P* = 0.211	*P* = 0.338
	Standing	6.1 ± 2.8	4.9 ± 2.5	4.8 ± 3.0	*P* = 0.149	*P* = 0.171	*P* = 0.943
RMSSD (ms)	Supine	**63.9** **±** **40.3**	**47.1** **±** **27.7**	**43.8** **±** **25.2**	*P* = 0.131	*P* = 0.065	*P* = 0.697
	Standing	**24.8** **±** 13.5 *	**23.2** **±** 16.8 *	**28.2** **±** 34.4 *	*P* = 0.747	*P* = 0.680	*P* = 0.562
RRI-30:15 ratio	Upon standing	1.5 ± 0.3	1.4 ± 0.3	1.4 ± 0.4	*P* = 0.166	*P* = 0.288	*P* = 0.879

*RESP* respiration, *BPsys* systolic blood pressure, *BPdia* diastolic blood pressure, *RRI* RR interval, *SD* standard deviation, *CV* coefficient of variance, *RMSSD* square-root-of-mean-squared-differences-of-successive-RRIs, *cpm* cycles per minute

An asterisk (*) indicates a significant difference (*P* < 0.05) between supine and standing values. Significant differences between groups are indicated in bold and underlined for the parameters, and in bold underlined italics for the *P*-values

**Table 2:** Mean values ± standard deviation of frequency domain parameters reflecting powers of autonomic modulation of RR intervals and blood pressure, and baroreflex sensitivity in supine and in standing positions in 20 patients with a history of mild TBI, 20 patients with a history of moderate or severe TBI (moderate–severe TBI) and 20 healthy controls

Parameter	Position	Healthy controls (*n* = 20)	Mild-TBI patients (*n* = 20)	Moderate–severe TBI patients (*n* = 20)	Mild TBI vs. controls	Moderate–severe TBI vs. controls	Mild TBI vs. moderate–severe TBI
LF-RRI powers (ms^2^)	Supine	2650.3 ± 4336.0	1881.2 ± 1983.0	1939.2 ± 1832.4	*P* = 0.841	*P* = 0.583	*P* = 0.534
	Standing	1319.8 ± 1292.7	1123.6 ± 1247.6	1725.7 ± 3056.0	*P* = 0.640	*P* = 0.478	*P* = 0.547
LFnu-RRI powers (%)	Supine	**49.6** **±** **14.3**	**61.2** **±** **15.9**	**71.4** **±** **8.9**	*P* = ***0.020***	*P* = ***0.000***	*P* = ***0.017***
	Standing	**80.8** **±** 12.5 *	**79.2** **±** 15.2 *	**83.2** **±** 13.9 *	*P* = 0.728	*P* = 0.369	*P* = 0.395
HF-RRI powers (ms^2^)	Supine	**1837.4** **±** **1935.3**	**1117.1** **±** **1232.6**	702.5 ± 691.0	*P* = 0.157	*P* = ***0.018***	*P* = 0.534
	Standing	**292.8** **±** 314.1 *	**313.3** **±** 466.8 *	515.9 ± 1248.2	*P* = 0.640	*P* = 0.277	*P* = 0.678
HFnu-RRI powers (%)	Supine	**50.4** **±** **14.3**	**38.8** **±** **15.9**	**28.6** **±** **8.9**	*P* = ***0.020***	*P* = ***0.000***	*P* = ***0.017***
	Standing	**19.2** **±** 12.5 *	**20.8** **±** 15.2 *	**16.8** **±** 13.9 *	*P* = 0.728	*P* = 0.369	*P* = 0.395
LF/HF-RRI ratios	Supine	**1.4** **±** **0.7**	**2.7** **±** **2.6**	**3.8** **±** **2.8**	*P* = ***0.072***	*P* = ***0.000***	*P* = **0.194**
	Standing	**8.2** **±** 9.5 *	**7.1** **±** 6.3 *	**11.3** **±** 11.1 *	*P* = 0.883	*P* = 0.369	*P* = 0.279
LF-BPsys powers (mmHg^2^)	Supine	**15.0** **±** **8.5**	14.4 ± 12.1	18.8 ± 14.8	*P* = 0.429	*P* = 0.799	*P* = 0.301
	Standing	**27.1** **±** 20.3 *	24.4 ± 25.8	35.5 ± 32.2	*P* = 0.214	*P* = 0.512	*P* = 0.089
HF-BPsys powers (mmHg^2^)	Supine	**1.8** **±** **1.2**	**2.1** **±** **1.4**	**2.1** **±** **1.9**	*P* = 0.602	*P* = 0.718	*P* = 0.862
	Standing	**7.4** **±** 1.3 *	**6.2** **±** 5.3 *	**10.5** **±** 13.6 *	*P* = 0.309	*P* = 0.738	*P* = 0.509
BRS	Supine	**13.8** **±** **8.3**	**11.1** **±** **4.8**	**10.8** **±** **5.8**	*P* = 0.461	*P* = 0.221	*P* = 0.583
	Standing	**6.2** **±** 3.6 *	**5.8** **±** 3.3 *	**5.6** **±** 5.7 *	*P* = 0.813	*P* = 0.369	p = 0.411

*RRI* RR interval, *BPsys* systolic blood pressure, *LF* low frequency, *LFnu* normalized LF powers, *HF* high frequency, *HFnu* normalized HF powers, *BRS* baroreflex sensitivity

An asterisk (*) indicates a significant difference (*P* < 0.05) between supine and standing values

Significant differences between groups are indicated in bold and underlined for the parameters, and in bold underlined italics for the *P*-values

For all our TBI patients (i.e., mild TBI and moderate–severe TBI patients), correlations of RRI, BPsys, BPdia and RESP and autonomic parameters with GCS scores at the time of injury, and with the interval since the injury are shown in Table 3. At supine rest, GCS scores did not correlate with any of the bio-signals or autonomic parameters (RRI-SD, RRI-CV, RMSSD, LF-RRI powers, LFnu-RRI powers, HF-RRI powers, HFnu-RRI powers, LF/HF-RRI ratios, LF-BPsys powers, HF-BPsys powers, BRS) (*P* > 0.05). However, upon orthostatic challenge, GCS scores correlated with standing values of normalized LF-RRI powers (*ρ* = 0.44, *P* = 0.013), and LF/HF-RRI ratios (*ρ* = 0.44, *P* = 0.013) and inversely correlated with standing values of normalized HF-RRI powers (*ρ* = −0.44, *P* = 0.013).

**Table 3:** Correlations of cardiovascular parameters with Glasgow Coma Scale (GCS) scores and with the interval since trauma in patients with a history of mild, moderate or severe TBI

Parameters	Position	Correlation with GCS in all TBI patients	Correlation with interval since trauma (months)		
			All TBI patients	Mild-TBI patients	Moderate–severe TBI patients
RESP (cpm)	Supine	*ρ* = –0.246, *P* = 0.175	*ρ* = 0.081, *P* = 0.621	*ρ* = 0.215, *P* = 0.363	*ρ* = 0.047, *P* = 0.845
	Standing	*ρ* = 0.005, *P* = 0.979	*ρ* = 0.107, *P* = 0.527	*ρ* = –0.015, *P* = 0.950	*ρ* = 0.363, *P* = 0.152
BPsys (mmHg)	Supine	*ρ* = –0.202, *P* = 0.269	*ρ* = –0.055, *P* = 0.736	*ρ* = –0.358, *P* = 0.121	*ρ* = –0.013, *P* = 0.957
	Standing	*ρ* = –0.161, *P* = 0.388	*ρ* = 0.091, *P* = 0.581	*ρ* = –0.146, *P* = 0.552	*ρ* = 0.181, *P* = 0.444
BPdia (mmHg)	Supine	*ρ* = –0.194, *P* = 0.288	*ρ* = –0.107, *P* = 0.511	*ρ* = –0.367, *P* = 0.112	*ρ* = –0.082, *P* = 0.731
	Standing	*ρ* = –0.274, *P* = 0.136	*ρ* = 0.074, *P* = 0.656	*ρ* = 0.009, *P* = 0.972	*ρ* = 0.102, *P* = 0.668
RRI (ms)	Supine	*ρ* = –0.085, *P* = 0.645	*ρ* = 0.160, *P* = 0.325	*ρ* = –0.044, *P* = 0.855	*ρ* = 0.302, *P* = 0.195
	Standing	*ρ* = 0.012, *P* = 0.946	*ρ* = 0.166, *P* = 0.307	*ρ* = 0.072, *P* = 0.762	*ρ* = 0.199, *P* = 0.401
RRI-SD (ms)	Supine	*ρ* = –0.029, *P* = 0.876	*ρ* **=** ***0.356***, *P* = ***0.024***	*ρ* **=** ***0.559***, *P* = ***0.010***	*ρ* = 0.138, *P* = 0.560
	Standing	*ρ* = 0.153, *P* = 0.404	*ρ* = 0.187, *P* = 0.247	*ρ* = 0.385, *P* = 0.094	*ρ* = 0.037, *P* = 0.877
RRI-CV (%)	Supine	*ρ* = 0.038, *P* = 0.836	*ρ* **=** ***0.315***, *P* = ***0.048***	*ρ* **=** ***0.594***, *P* = ***0.006***	*ρ* = 0.041, *P* = 0.862
	Standing	*ρ* = 0.162, *P* = 0.375	*ρ* = 0.129, *P* = 0.429	*ρ* = 0.319, *P* = 0.171	*ρ* = 0.031, *P* = 0.897
RMSSD (ms)	Supine	*ρ* = –0.071, *P* = 0.698	*ρ* **=** **0.329,** *P* = **0.038**	*ρ* **=** **0.430,** *P* = **0.058**	*ρ* = 0.197, *P* = 0.405
	Standing	*ρ* = 0.006, *P* = 0.976	*ρ* = 0.129, *P* = 0.429	*ρ* = 0.214, *P* = 0.366	*ρ* = 0.076, *P* = 0.750
RRI-30:15 ratio	Standing	*ρ* = –0.050, *P* = 0.856	*ρ* = 0.036, *P* = 0.828	*ρ* = –0.065, *P* = 0.786	*ρ* = 0.136, *P* = 0.567
LF-RRI powers (ms^2^)	Supine	*ρ* = –0.094, *P* = 0.609	*ρ* **=** ***0.365***, *P* = ***0.021***	*ρ* **=** ***0.532***, *P* = ***0.016***	*ρ* = 0.148, *P* = 0.533
	Standing	*ρ* = 0.136, *P* = 0.458	*ρ* = 0.136, *P* = 0.403	*ρ* = 0.359, *P* = 0.120	*ρ* = –0.059, *P* = 0.806
LFnu-RRI powers (%)	Supine	*ρ* = –0.148, *P* = 0.419	*ρ* = 0.110, *P* = 0.499	*ρ* = 0.077, *P* = 0.748	*ρ* = 0.120, *P* = 0.615
	Standing	*ρ* **=** ***0.436***, *P* = ***0.013***	*ρ* = –0.107, *P* = 0.511	*ρ* = 0.065, *P* = 0.787	*ρ* = –0.321, *P* = 0.167
HF-RRI powers (ms^2^)	Supine	*ρ* = 0.046, *P* = 0.803	*ρ* **=** ***0.280***, *P* = ***0.080***	*ρ* **=** ***0.417***, *P* = ***0.068***	*ρ* = 0.168, *P* = 0.479
	Standing	*ρ* = –0.149, *P* = 0.416	*ρ* = 0.134, *P* = 0.411	*ρ* = 0.150, *P* = 0.527	*ρ* = 0.164, *P* = 0.490
HFnu-RRI powers (%)	Supine	*ρ* = 0.148, *P* = 0.419	*ρ* = –0.110, *P* = 0.499	*ρ* = –0.077, *P* = 0.748	*ρ* = –0.120, *P* = 0.615
	Standing	*ρ* **=** **–** ***0.436***, *P* = ***0.013***	*ρ* = 0.107, *P* = 0.511	*ρ* = –0.065, *P* = 0.787	*ρ* = 0.321, *P* = 0.167
LF/HF-RRI ratios	Supine	*ρ* = –0.238, *P* = 0.189	*ρ* = 0.128, *P* = 0.433	*ρ* = 0.074, *P* = 0.758	*ρ* = 0.134, *P* = 0.573
	Standing	*ρ* **=** ***0.436***, *P* = ***0.013***	*ρ* = –0.107, *P* = 0.511	*ρ* = 0.065, *P* = 0.787	*ρ* = –0.321, *P* = 0.167
LF-BPsys powers (mmHg^2^)	Supine	*ρ* = 0.026, *P* = 0.889	*ρ* = 0.017, *P* = 0.917	*ρ* = 0.382, *P* = 0.097	*ρ* = –0.378, *P* = *P* = 0.100
	Standing	*ρ* = 0.164, *P* = 0.377	*ρ* = –0.204, *P* = 0.213	*ρ* = –0.107, *P* = 0.663	*ρ* **=** **–** ***0.451***, *P* = ***0.046***
HF-BPsys powers (mmHg^2^)	Supine	*ρ* = –0.008, *P* = 0.967	*ρ* = 0.016, *P* = 0.924	*ρ* = 0.141, *P* = *P* = 0.552	*ρ* = –0.054, *P* = 0.821
	Standing	*ρ* = –0.249, P = 0.177	*ρ* = –0.233, *P* = 0.153	*ρ* = –0.475, *P* = 0.040	*ρ* = 0.160, *P* = 0.502
BRS	Supine	*ρ* = –0.035, *P* = 0.848	*ρ* **=** ***0.296***, *P* = ***0.063***	*ρ* = 0.334, *P* = 0.150	*ρ* = 0.338, *P* = 0.145
	Standing	*ρ* = 0.049, *P* = 0.792	*ρ* = 0.246, *P* = 0.132	*ρ* = 0.405, *P* = 0.085	*ρ* = 0.202, *P* = 0.392

*RESP* respiration, *BPsys* systolic blood pressure, *RRI* RR interval, *SD* standard deviation, *CV* coefficient of variance, *RMSSD* square root of mean squared differences of successive RRIs, *LF* low frequency, *LFnu* normalized LF powers, *HF* high frequency, *HFnu* normalized HF powers, *BRS* baroreflex sensitivity

Significant correlations are indicated in bold and italic fonts

Only in the patients with a history of mild TBI did we find significant direct correlations between the interval since the trauma and supine resting values of RRI-SD (*ρ* = 0.56, *P* = 0.010), RRI-CV (*ρ* = 0.59, *P* = 0.006), and LF-RRI powers (*ρ* = 0.365, *P* = 0.021), and a tendency for supine RMSSD (*ρ* = 0.43, *P* = 0.058) and supine HF-RRI powers (*ρ* = 0.42, *P* = 0.068). In the moderate–severe TBI patients, only standing values of LF-BPsys powers correlated inversely with the interval since the trauma (*ρ* = −0.45, *P* = 0.046).

## Discussion

Our study shows a correlation between the severities of the initial TBI and of cardiovascular autonomic dysregulation both under resting condition and upon orthostatic challenge, as well as a tendency towards recovery of autonomic function with the time interval post-injury.

Compared to healthy controls, patients with a history of moderate–severe as well as mild TBI have increased sympathetic and decreased parasympathetic cardiovascular autonomic modulation already at rest, as shown by significantly higher systolic BP values in patients after moderate–severe TBI and higher LFnu-RRI powers, lower parasympathetically mediated HFnu-RRI powers and lower LF/HF-RRI ratios in both patient groups than in the controls (Tables 1, 2).

To some extent, the current results confirm our previous findings in patients with a history of mild TBI [20] who showed reduced cardiovascular autonomic modulation with a shift towards increased sympathetic modulation already at rest, and additionally had compromised baroreflex function upon standing-up [20]. However, our current data additionally show an association between the severity of the past trauma and the autonomic impairment, already at rest. Resting autonomic dysfunction was more prominent in the moderate–severe TBI patients than in mild-TBI patients while mild-TBI patients still had altered autonomic modulation and more sympathetic modulation than had the controls.

In the mild-TBI patients, sympathetic modulation was significantly higher and parasympathetic modulation was significantly lower (*P* = 0.02) than in the healthy controls. In the patients with moderate–severe TBI, the increase in sympathetic modulation and decrease in parasympathetic modulation still was more pronounced than in the mild-TBI patients (*P* = 0.017).

Upon orthostatic challenge, both patient groups had compromised baroreflex function. When standing-up, post-TBI patients could not significantly increase sympathetically mediated BP modulation, i.e., LF-BPsys powers. However, only the patients with a history of moderate-to-severe TBI were unable to significantly withdraw parasympathetic cardiac modulation as shown by their non-significant changes in RRI-HF powers upon standing-up (Table 2). In contrast, patients after mild TBI could not adequately raise their sympathetically mediated LF-BPsys modulation but still decreased HF-RRI modulation similarly to the controls.

In addition, standing-up unveiled a direct correlation between the initial trauma severity, as assessed by GCS scores, and the parameters reflecting sympathetic modulation, LFnu-RRI powers and LF/HF ratios, as well as an inverse correlation between GCS scores and HFnu-RRI powers reflecting parasympathetic modulation. The correlations show that a more severe trauma yields a more pronounced inability to mount sympathetic activation and to withdraw parasympathetic modulation upon baroreflex unloading even months to years after the trauma.

These findings are at par with studies showing that more severe TBI causes more severe neurological, neurodegenerative, neuroendocrine and psychiatric sequelae, including more severe cognitive impairment [47, 56], a higher incidence of post-traumatic seizures [2, 33], higher risk of developing neurodegenerative disorders such as Alzheimer’s disease [41] or Parkinson’s disease [6], and greater prevalence of hypopituitarism [5, 46].

As already postulated in our previous studies [19–21], we assume that TBI-related pathologies alter CAN function [3] and thus induce cardiovascular autonomic changes. Various pathologies have been described in patients even years after TBI, including diffuse cerebral volume loss [15, 30], white matter injuries [11, 24, 45] and gray matter de-afferentation [24, 37], located in areas such as the uncinate fasciculus, genu of the corpus callosum, inferior longitudinal fasciculus, cingulum bundle, and infratentorial brainstem [24, 30, 37, 45], i.e., brain regions that contribute to the CAN and might cause cardiovascular autonomic dysregulation if injured [20, 40].

Neuroimaging studies have also shown correlations between TBI severity and the severity of MRI changes [48]. In patients with a history of mild-to-severe TBI, Gale et al. found associations between lower GCS scores, i.e., more severe TBI, and a more pronounced decrease in gray matter concentration 1-year post-injury [15]. In 69 chronic-phase TBI patients with all grades of TBI severity, Levine et al. observed more severe parenchymal volume loss in moderate–severe than in mild-TBI patients who still had significantly less brain volume than the healthy control persons [30]. These findings not just confirm common knowledge that more severe TBI causes more severe brain lesions, but also support our conclusion that more severe TBI may result in more severe CAN dysfunction and thus cardiovascular autonomic dysregulation.

Only in patients with a history of mild TBI, did we see a recovery of the overall and the sympathetic cardiac autonomic modulation with increasing intervals since the trauma, as evidenced by the correlations between RRI-SD, RRI-CV and LF-RRI powers with the interval since the trauma (Table 3). Probably, the correlations were rather limited due to the wide range of the interval since the TBI in our 40 patients and due to the small sample size of our study participants. Still, the data show a trend towards a recovery of cardiac autonomic modulation with time.

The finding of cardiovascular autonomic dysfunction in patients with a history of TBI might have prognostic relevance. Our finding of more severe cardiovascular autonomic dysfunction in patients with a history of moderate–severe TBI than in patients with a history of mild TBI might in part explain the higher long-term mortality rates in patients who suffered moderate–severe TBI than in patients who had mild TBI [12, 13]. In a Swedish population study lasting for 41 years, patients who had survived a TBI for at least 6 months had 3-times higher mortality rates than the general population; yet, among patients who had experienced a moderate–severe TBI mortality rates were 4.5-times higher than in the general population [13]. Hendricks et al. also showed the prognostic value of autonomic dysfunction in a study of 79 patients with acute severe TBI [17]. Those 11.8% patients who had autonomic dysregulation with increased respiratory rate, HR, BP, temperature, muscle, sweating, etc., during the acute phase of the trauma tended to have a poorer outcome 6 months after trauma [17].

The autonomic changes seen in our patients at rest and upon standing were subtle and none of our patients had any signs or symptoms of autonomic dysfunction at the time of autonomic testing. While the observed changes indicate that there is a central autonomic dysfunction even years after the TBI, the clinical effects of the changes were mild and any medication such as treatment with a beta-adrenergic blocker might have more effects on autonomic control than clinical observation and training [14, 28, 29, 31]. Hence, we informed our patients about the subtle changes and suggested that they should perform mild and continuous endurance training and aerobic exercise which may shift the observed slight sympathetic predominance towards more parasympathetic activity and may thus improve autonomic balance [14, 31] and baroreflex function [28, 29]. These changes might have beneficial effects on the long-term prognosis [9, 25, 54]. In addition, we informed the patients about the need to seek medical advice in case of any overt autonomic changes such as dizziness or nausea upon standing up, palpitations or tachycardia, and we recommended follow-up examinations.

## Study limitations

Clinical parameters other than the GCS scores, for example the duration of coma, the occurrence of secondary complications after TBI, the presence of diffuse axonal lesions, might also be associated with the cardiovascular autonomic dysfunction. Hendricks et al. found autonomic dysfunction with increased respiratory rate, HR, BP, temperature, muscle, sweating, etc., in 11.8% of 76 patients during the acute first 1–22 days after severe TBI [17]. Those patients who experienced dysautonomia had longer periods of coma and mechanical ventilation, and higher prevalence of diffuse axonal lesions [17]. It is a limitation of our study that we did not evaluate any correlation of these parameters with autonomic parameters. However, our retrospective analysis of data at the time of injury implied that some data sets were incomplete. Moreover, our sample size was too small for multiple statistical analyses of correlations between autonomic parameters and parameters such as initial coma duration or time of mechanical ventilation. Yet, we could not easily increase the number of study participants as we only included patients with a history of TBI who had reached a level of independence in activities of daily living with reduced capacity for former work, i.e., GOSE values of 6 and above [17, 53], who had no overt autonomic dysfunction at the time of our study, and who, moreover, were on no medication or had any diseases that might influence autonomic function.

While the correlation between cardiovascular autonomic parameters and the interval since the initial trauma shows a partial recovery of autonomic cardiac regulation with increasing time since the trauma, we have not performed longitudinal repeat-measurements of cardiovascular autonomic parameters in our patients. Such follow-up assessments might further elucidate the recovery process indicated by the correlations between improving autonomic parameters and increasing intervals since the trauma.

## Conclusions

In summary, our data support our hypothesis that patients with a history of moderate or severe TBI have more prominent cardiovascular autonomic dysregulation at rest and upon orthostatic challenge than do patients with a history of mild TBI. These differences may be due to more prominent central autonomic network alteration in patients with more severe brain injuries.
